# Characterization of complex structural variation in the *CYP2D6-CYP2D7-CYP2D8* gene loci using single-molecule long-read sequencing

**DOI:** 10.3389/fphar.2023.1195778

**Published:** 2023-06-22

**Authors:** Amy J. Turner, Ashley D. Derezinski, Andrea Gaedigk, Mark E. Berres, David B. Gregornik, Keith Brown, Ulrich Broeckel, Gunter Scharer

**Affiliations:** ^1^ RPRD Diagnostics LLC, Milwaukee, WI, United States; ^2^ Children’s Mercy Research Institute, Kansas City, MO, United States; ^3^ Biotechnology Center, University of Wisconsin Madison, Madison, WI, United States; ^4^ Children’s Minnesota, Minneapolis, MN, United States; ^5^ Jumpcode Genomics, San Diego, CA, United States

**Keywords:** *CYP2D6*, pharmacogenetics, PCR-free, clinical testing, CRISPR, single-molecule long-read sequencing, precision medicine

## Abstract

Complex regions in the human genome such as repeat motifs, pseudogenes and structural (SVs) and copy number variations (CNVs) present ongoing challenges to accurate genetic analysis, particularly for short-read Next-Generation-Sequencing (NGS) technologies. One such region is the highly polymorphic *CYP2D* loci, containing *CYP2D6,* a clinically relevant pharmacogene contributing to the metabolism of >20% of common drugs, and two highly similar pseudogenes, *CYP2D7* and *CYP2D8*. Multiple complex SVs, including *CYP2D6/CYP2D7*-derived hybrid genes are known to occur in different configurations and frequencies across populations and are difficult to detect and characterize accurately. This can lead to incorrect enzyme activity assignment and impact drug dosing recommendations, often disproportionally affecting underrepresented populations. To improve *CYP2D6* genotyping accuracy, we developed a PCR-free CRISPR-Cas9 based enrichment method for targeted long-read sequencing that fully characterizes the entire *CYP2D6-CYP2D7-CYP2D8* loci. Clinically relevant sample types, including blood, saliva, and liver tissue were sequenced, generating high coverage sets of continuous single molecule reads spanning the entire targeted region of up to 52 kb, regardless of SV present (*n* = 9). This allowed for fully phased dissection of the entire loci structure, including breakpoints, to accurately resolve complex *CYP2D6* diplotypes with a single assay. Additionally, we identified three novel *CYP2D6* suballeles, and fully characterized 17 *CYP2D7* and 18 *CYP2D8* unique haplotypes. This method for *CYP2D6* genotyping has the potential to significantly improve accurate clinical phenotyping to inform drug therapy and can be adapted to overcome testing limitations of other clinically challenging genomic regions.

## 1 Introduction

Precisely determining allele structure and phased diplotype assignment is of particular importance in clinical testing, including pharmacogenetics (PGx). Complex regions in the human genome, including repeat motifs, pseudogenes, and structural (SVs) and copy number variations (CNVs) have presented substantial challenges for both research and clinical analyses, particularly with short-read Next-Generation-Sequencing (NGS) technologies (Chaisson et al., 2015; Nofziger and Paulmichl, 2018).

Recent studies have shown advancements in using long-read sequencing (LRS) for mapping and phasing of structural variation (Shi et al., 2016; Cretu Stancu et al., 2017; Leung et al., 2022; Zhou et al., 2022). The long reads generated in LRS allow for direct SNP and SV/CNV phasing. Methodologies such as SMRT long-read (Pacific Biosciences) and nanopore (Oxford Nanopore Technologies; ONT) sequencing can overcome some of the limitations of complex variation analysis (Yang et al., 2017; Mantere et al., 2019; Erdmann et al., 2023), allowing for accurate characterization of SV/CNV with high sensitivity in as little as 11–16X coverage (Cretu Stancu et al., 2017). Furthermore, applications using long read targeted sequencing methods can analyze larger numbers of samples at increased sequencing depth, while reducing the cost of downstream data analysis and storage burden, compared to whole-exome (WES) and genome sequencing (WGS) (Bewicke-Copley et al., 2019). However, challenges still remain, including the analysis of regions with nested or multiple overlapping rearrangements or those with highly similar psuedogenes (Stephens et al., 2018; Mai et al., 2019; Amarasinghe et al., 2020).

The utilization of CRISPR-Cas9 genomic enrichment has allowed for the development of targeted PCR-free LRS approaches, but has been limited by the optimal target fragment size of approximately 25 kb or less, and still relies on multiple overlapping read alignments and in-depth computational analysis for larger regions (Huddleston et al., 2017; Gilpatrick et al., 2020). To address the current targeted LRS limitations, we developed a CRISPR-Cas9 based, PCR-free approach which allows for the enrichment of continuous segments greater than 50 kb for ONT nanopore sequencing. Starting with DNA extraction, the benchtop workflow requires approximately 8 hours, and when coupled with sequencing takes less than 36 hours to complete, depending on the desired read depth.

To assess the clinical potential of our approach, we evaluated its performance on one of the most clinically relevant and challenging pharmacogenes, *CYP2D6*, which contributes to the metabolism of over 20% of prescribed drugs (Saravanakumar et al., 2019). The extremely polymorphic *CYP2D* loci confounds traditional genotyping platforms due to its large size and complex structure, which includes up- and downstream repetitive regions, the *CYP2D6* gene, and two highly similar pseudogenes, *CYP2D7* and *CYP2D8* (Nofziger et al., 2020).

Multiple *CYP2D6-2D7* hybrid gene structures, full *CYP2D6* gene duplications and deletions with variable and often poorly defined breakpoints are routinely found. These CNVs and SVs occur with varying frequencies across populations and may not be included in, or can interfere with, testing platforms (Gaedigk et al., 1991; Steijns and Van Der Weide, 1998; Gaedigk, 2013; Hicks et al., 2014; Scantamburlo et al., 2017; Del Tredici et al., 2018; Gaedigk et al., 2018; Nofziger and Paulmichl, 2018; Nofziger et al., 2020). Additionally, SNPs, particularly those in the *CYP2D7* pseudogene, can also interfere with *CYP2D6* genotyping (Gaedigk et al., 2015; Numanagić et al., 2015; Riffel et al., 2015). To date, over 160 unique *CYP2D6* haplotypes (annotated using star (*) allele nomenclature) and numerous CNVs and hybrid structures in various arrangements have been described by the Pharmacogene Variation (PharmVar) Consortium (Gaedigk et al., 2021; Nofziger et al., 2020). Accurate clinical genotyping is critical in assigning metabolizer status as many of these haplotypes have altered enzyme function, consequently impacting drug metabolism and individual response to drug therapy (Iversen et al., 2022).

## 2 Methods

**FIGURE 1:**
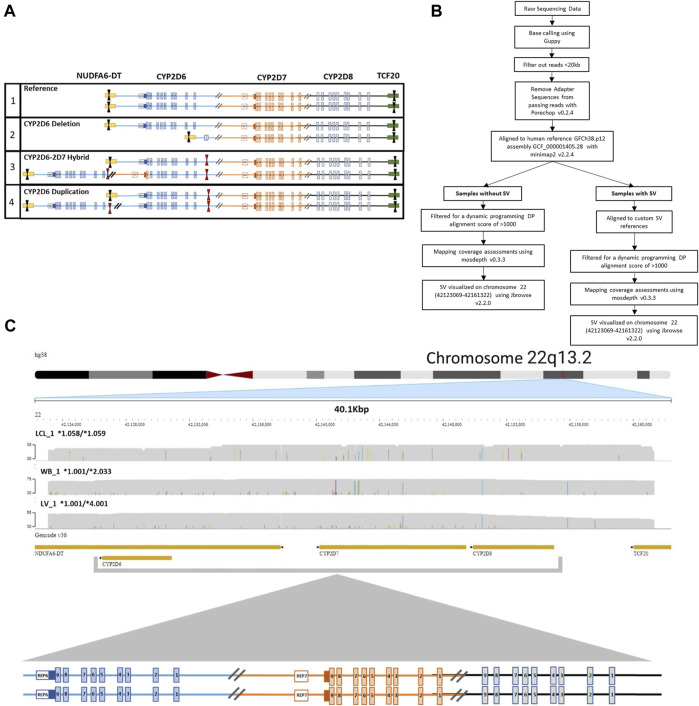
CRISPR-CAS9 Enrichment of region of interest. **(A)** Visualization of crRNA target cut sites on (1) reference *CYP2D6-2D7-2D8* loci, (2) CYP2D6*5 full gene deletion, (3) CYP2D6*36+*10 tandem containing a hybrid gene, and (4) *CYP2D6* duplication of two identical gene copies. Black arrows indicate crRNAs selected for use. Red arrows indicate unselected crRNA within the region of interest that could lead to the introduction of multiple cut sites in samples with a copy number or structural variant (SV/CNV). **(B)** Analysis workflow for samples with and without an SV. **(C)** Representative JBrowse2 alignments of 38 kb long reads to hg38, generated from samples without an SV. Sample sources include lymphoblast cell line (LCL_1, top), whole blood (WB_1, middle), and liver tissue (LV_1, bottom). *CYP2D6* diplotypes are annotated as *alleles for each sample. The crRNA cut sites are located in *NDUFA6-DT* (downstream of *CYP2D6*) and *TCF20* (upstream of *CYP2D8*).

To ensure coverage of all relevant SVs and CNVs when performing targeted enrichment, the entire *CYP2D6-2D7-2D8* loci was captured. Structurally complex samples (i.e., duplication and hybrid alleles) may contain multiple on-target cut sites in duplicated regions or may be lost in samples with deletions within the loci, potentially interfering with accurate phasing and CN calling. To avoid this, we designed one set of CRISPR RNAs (crRNA) that target outside the entire loci, ranging up to 52 kb in size, depending on SV/CNV present. Tiling of multiple sets of crRNAs can generate unnecessary additional fragments within the loci, which requires computational phasing and can confound accurate haplotype and diplotype assignments. The designed 3′ and 5′ crRNAs encompass all three genes and relevant breakpoints, enabling direct haplotype phasing regardless of structural composition (Figure 1A).

### 2.1 Guide RNA design and validation

To capture the full *CYP2D6-2D7-2D8* loci, crRNAs were designed to target the 3′ and 5′ ends of the region NC_000022.11:42,122,008-42,161,558 (*Homo sapiens* chromosome 22, GRCh38.p14, assembly GCF_000001405.40), which were synthesized by Integrated DNA Technologies (IDT) (Supplementary Table S1; Supplementary Figure S1). The crRNAs were selected based on predicted on-target potential and off-target risk, in which those with the highest on-target potential and lowest off-target risk were selected. Guide RNAs (gRNAs) were generated through annealing of each of the crRNAs with trans-acting CRISPR RNA (tracrRNA).

To first assess crRNA performance, PCR amplicons were generated that spanned the 5′ and 3′ cut sites (Amplicons A and C, Supplementary Figure S1A). After amplification, PCR products were purified using AMPure XP beads (Beckman Coulter) per manufactures recommendations. Purified amplicons were quantified using the Invitrogen™ Qubit™ 2.0 Fluorometer with the Qubit™ Broad Range Assay Kit.

Cutting efficiency of the gRNAs were assessed by the formation of Cas9 complex and cutting of long range PCR (XL-PCR) amplicons generated to contain the predicted cut site using XL-PCR generated double stranded amplicons (Supplementary Figure S1). The XL-PCR was performed using primers listed in Supplementary Table S1 and TaKaRa LA Taq DNA Polymerase Hot-Start Version kit (Takara). Thermal cycling conditions are described in Supplementary Table S2.

Reaction results were compared to uncut amplicons and control reactions of amplicons not containing the target cut site. The gRNAs with the highest cutting efficiency in amplicons were selected for additional validation using high molecular weight (HMW) DNA.

The HMW DNA was cut with the gRNA + CRISPR-Cas9 complex, and then subsequent XL-PCR was performed to generate amplicons spanning the 3′ and 5′ cut sites (Amplicons A and C) as well as an untargeted region containing *CYP2D6* between the cut sites (Amplicon B). Cutting efficiency of the HMW DNA was determined by comparing the XL-PCR amplicons containing the cut sites to the untargeted region (Supplementary Figure S1). The 3′ and 5′ gRNAs with the highest percent of cutting at the target sites were selected. The overall design and gRNA validation process is described in Supplementary Figure S1C.

### 2.2 Sample selection

Sample types were selected based on their clinical relevance to PGx testing. These included Lymphoblastoid cell lines (LCL) purchased from the Coriell Institute for Medical Research, whole blood, saliva, and liver tissue.

Whole blood was collected in EDTA-tubes which were stored at 4°C until time of extraction. Saliva was collected using the DNAgenoTeK^®^ Oragene™ OG-500 kit and stored at room temperature per manufacturer recommendations until DNA extraction. The liver tissue sample was obtained from the Eunice Kennedy Shriver National Institute of Child Health and Human Development (NICHD)-supported tissue retrieval program at the Brain and Tissue Bank for Developmental Disorders at the University of Maryland (now the University of Maryland Brain and Tissue Bank). Use of the tissue sample was classified as non-human subjects research by the Children’s Mercy Pediatric Institutional Review Board.

Samples tested had been previously genotyped using other current testing methods and had either unresolved diplotypes or required multiple assays to initially determine accurate copy number state. The blood, saliva, and liver tissue samples had been previously genotyped on the ThermoFisherScientific PharmacoScan™ Array and LCL samples were genotyped as part of the GeT-RM studies (Pratt et al., 2016; Gaedigk et al., 2019). Study samples, sample source, and known/previous genotypes are described in Tables 1, 2.

**TABLE 1:** Study samples without structural variation.

Sample ID	Sample source	Known *CYP2D6* diplotype	Detected *CYP2D6* diplotype
LCL_1 (GM19213)^a^ ^,^ ^b^ ^,^ ^c^	Lymphoblastoid cell line	*1/*1	*1.058/*1.059
WB_1^b^	Whole blood	*1/*2 or *34/Unknown	*1.001/*2.033
LV_1^b^ ^,^ ^d^	Liver Tissue	*1/*4 or *1/*68+*4	*1.001/*4.001

Per PharmVar annotations, annotations for multiplications reflect their position on the allele (the most 5′ gene copy (or gene copy in the “duplicated” position) shown first.

^a^ Previously described genotypes derived from Get-RM (Gaedigk et al., 2019; Wang et al., 2022).

^b^ PharmacoScan.

^c^ Sample run twice as technical replication. Coriell IDs are in () where applicable.

^d^ Sanger sequencing.

The fully characterized genotypes generated with the CRISPR-Cas9 LRS sequencing are shown in the Detected *CYP2D6* Genotype column.

The italic numbers are the *CYP2D6* star allele diplotypes.

**TABLE 2:** Study samples with structural variation.

Sample ID	Sample source	Known *CYP2D6* diplotype	Detected *CYP2D6* diplotypes
LCL_2 (GM18959)^a^ ^,^ ^b^	Lymphoblastoid cell line	*2/*36+*10	*2.001/*36+*10.001
LCL_3 (GM06984)^a^ ^,^ ^b^	Lymphoblastoid cell line	*4/*68+*4	*4.001/*68+*4.001
LCL_4 (GM18855)^a^ ^,^ ^b^	Lymphoblastoid cell line	*1/*5	*1.045/*5.001
LCL_5 (GM19790)^a^ ^,^ ^b^	Lymphoblastoid cell line	*1/*13+*2	*1.001/*13+*2.001
LCL_6 (GM19109)^a^ ^,^ ^b^ ^,^ ^c^	Lymphoblastoid cell line	*2x2/*29	*2.001x2/*29.001
SA_1^b^	Saliva	*1/*68+*4	*1.037/*68+*4.001

Per PharmVar annotations, annotations for multiplications reflect their position on the allele [the most 5′ gene copy (or gene copy in the ‘duplicated’ position)] shown first.

^a^ Known genotypes derived from Get-RM (Gaedigk et al., 2019; Wang et al., 2022).

^b^ PharmacoScan. Coriell IDs are in () where applicable The fully characterized genotypes generated with the CRISPR-Cas9 LRS sequencing are shown in the Detected *CYP2D6* Genotype column.

^c^ Sample run twice as technical replication.

The italic numbers are the *CYP2D6* star allele diplotypes.

### 2.3 HMW DNA extraction

All samples, regardless of sample type or collection method, were extracted for HMW DNA using extraction methods designed for the specific sample type. The LCL (*n* = 6) and whole blood (*n* = 1) samples were extracted using New England Biolabs Monarch^®^ Genomic High Molecular Weight DNA Extraction Kit for cells and whole blood, following the protocol provided by the manufacturer and using the recommended sample standard input amounts. Similarly, liver tissue (*n* = 1) was extracted using New England Biolabs Monarch^®^ HMW Extraction kit for tissue using the manufacturer provided protocol. DNA was extracted from saliva (*n* = 1) using the DNAgenoTeK^®^ PrepIT.L2P extraction kit also following the protocol provided by the manufacturer. All HMW DNA samples were quantified using the Invitrogen™ Qubit™ 2.0 Fluorometer with the Qubit™ Broad Range Assay Kit to ensure adequate amounts of DNA for library preparation (7.5 µg HMW DNA). If DNA was not solubilized fully at room temperature samples were heated to 30°C for up to 1 hour. An additional quantification was done after heating to confirm the quantities.

### 2.4 Library preparation and sequencing on MinION

Libraries for sequencing were prepared from 7.5 µg of purified HMW DNA using the Oxford Nanopore Technologies Cas9 sequencing kit (SQK-CS9109) as recommended by the manufacturer, except for the tiling of gRNAs, which was omitted due to the repetitive and complex nature of the loci. Input amounts of 5µg, 7.5 µg, and 10 µg of DNA were tested, with no improved sequencing quality or depth between 7.5 µg and 10 µg. The optimized gRNAs (Supplementary Table S1) which included one 3′ and one 5′ targeting gRNA, were used to perform the cutting reactions. To ensure the HMW DNA was in solution, samples were heated to 30°C for at least 30 min before library preparation. Completed libraries were subsequently loaded onto a MinION v9.4.1 flow cell and sequenced on a MinION device utilizing MinKNOW control software, per manufactures instructions (Oxford Nanopore Technologies). If library loading resulted in less than 40% active sequencing pores, the run was paused, and additional library material was loaded. Sequencing continued for a minimum of 24 hours at default voltage (−180 mV) and Qscore threshold of seven. Samples LCL_1 and LCL_6 were each prepared and sequenced multiple times as technical replicates (*n* = 2 per sample).

### 2.5 Data analysis

Base calling of the raw nanopore reads was performed with Guppy version 6.0.1 + 652ffd1 with the “super accuracy” (SUP) model dna_r9.4.1_450bps_sup. Adapter sequences from passing reads were removed with Porechop v0.2.4, filtered from reads <20 kb and aligned to human reference GRCh38.p12, assembly GCF_000001405.38 with minimap2 v2.24. Mapping coverage was assessed using mosdepth v0.3.3 after filtering for a dynamic programming (DP) alignment score of >1000. Structural variants across an approximately 38 kb region of chromosome 22 (42123069-42161322), which includes *CYP2D6, CYP2D7*, *CYP2D8* were visualized with JBrowse 2 (v2.2.0).

The accurate alignment and visualization of samples with SV/CNV required the development of two custom reference tracks for different duplication and tandem hybrid arrangements. Samples were aligned to each refence sequence based on the nature of the 5′ region of *CYP2D6* and the presence or absence of a *CYP2D7* like 1.56 kb spacer (Supplementary Figure S1). Custom reference tracks developed for samples with specific SVs were used also for direct haplotyping of each allele in the tandem arrangements, which previously has relied on computational phasing. The genotype and haplotype results were then compared to existing data generated from other platforms (Pratt et al., 2016; Gaedigk et al., 2019). Variant calling was performed as previously described (Liau et al., 2019; Wang et al., 2022). All alignments were further viewed using the Integrative Genomics Viewer v.2.9.2 (IGV) aligning to the human GRCh38 reference genome to confirm diplotype calls (Thorvaldsdottir et al., 2013).

## 3 Results

Assay performance was assessed on nine samples from varying clinical types, with previously generated genotype data. This included three samples known to lack any SV/CNV (Table 1) and six samples with known SV/CNV (Table 2). All samples, regardless of DNA source or structural complexity, generated sufficient numbers of alignable reads for analysis (minimum >35 reads per sample) (Cretu Stancu et al., 2017; Mantere et al., 2019), which singularly spanned the entire *CYP2D6-2D7-2D8* loci (Supplementary Figure S2). Reads that did not capture the entire targeted region, as defined by the 3′ and 5′ targeted sites, were excluded from analysis. Single reads greater than 25 kb with PHRED-scaled Qscores averaging >14 had enrichment for the targeted region with average read depth >150X (Table 1).

In our study we included blood, saliva, liver tissue, and LCL cell lines to investigate any assay variability based on sample source or collection method. In addition to sample quantity, it is critical to obtain high quality HMW DNA to generate continuous reads through the entire loci, which can vary in size from 25 kb up to 52 kb depending on the *CYP2D6* SV/CNV present. Sufficient quantity and quality of HMW DNA for CRISPR-Cas-Based enrichment was obtained from all samples, regardless of collection method or source.

### 3.1 *CYP2D6* analysis in samples without SV/CNV

Human genome reference (GRCh38p.15; Chr22:42123054-42161339) aligned reads from the three samples without SV/CNV, LCL_1, WB_1 and LV_1 (Table 1) resulted in continuous alignments of approximately 38 kb (Figure 1C) covering the entire *CYP2D6-2D7-2D8* loci, demonstrating the successful enrichment of the targeted region. Phased variant calling and haplotype assignment of these samples (Supplementary Table S3) found 100% concordance with existing genotype data for regions in common between genotyping platforms.

For sample WB_1, the previous PharmacoScan™ analysis was unable to resolve phasing of the haplotypes, generating multiple possible core *allele calls (*1/*2 or *34/Unknown). Full coverage of the targeted region with continuous long reads allowed for the complete phased haplotype resolution of sample WB_1 and confirmed the *1/*2 call. GeT-RM genotyping of sample LCL_1 (Pratt et al., 2016; Gaedigk et al., 2019) previously reported a *CYP2D6* genotype call of *1/*1, which was concordant with our results for both technical replicates of the sample. Analysis of our data from sample LV_1 confirmed the pervious PharmacoScan™ genotyping assignment of *1/*4, which contradicted with the formation of an XL-PCR amplicon that was generated for Sanger sequencing, which suggested the presence of a SV/CNV, possibly a hybrid allele such as CYP2D6*68+*4.

Our phased variant genotype calling provided further resolution of the specific suballeles present in all three samples, as shown in Table 1. The suballeles (e.g. *2.033) of a core allele (e.g. *2) must contain the core allele defining sequence variants and have additional sequence variation present (Yang et al., 2017). Of note, in samples LCL_1 and WB_1 we identified three novel suballeles that had been either missed or ambiguously called previously on the other platforms. These novel suballeles were submitted to the PharmVar and have now been designated as CYP2D6*1.058, *1.059 and *2.033. Additionally, on the novel *2.033 suballele we identified a SNP (4882A>G (rs267608272)) previously only reported in CYP2D6*35.

### 3.2 *CYP2D6* analysis in samples with SV/CNV

**FIGURE 2:**
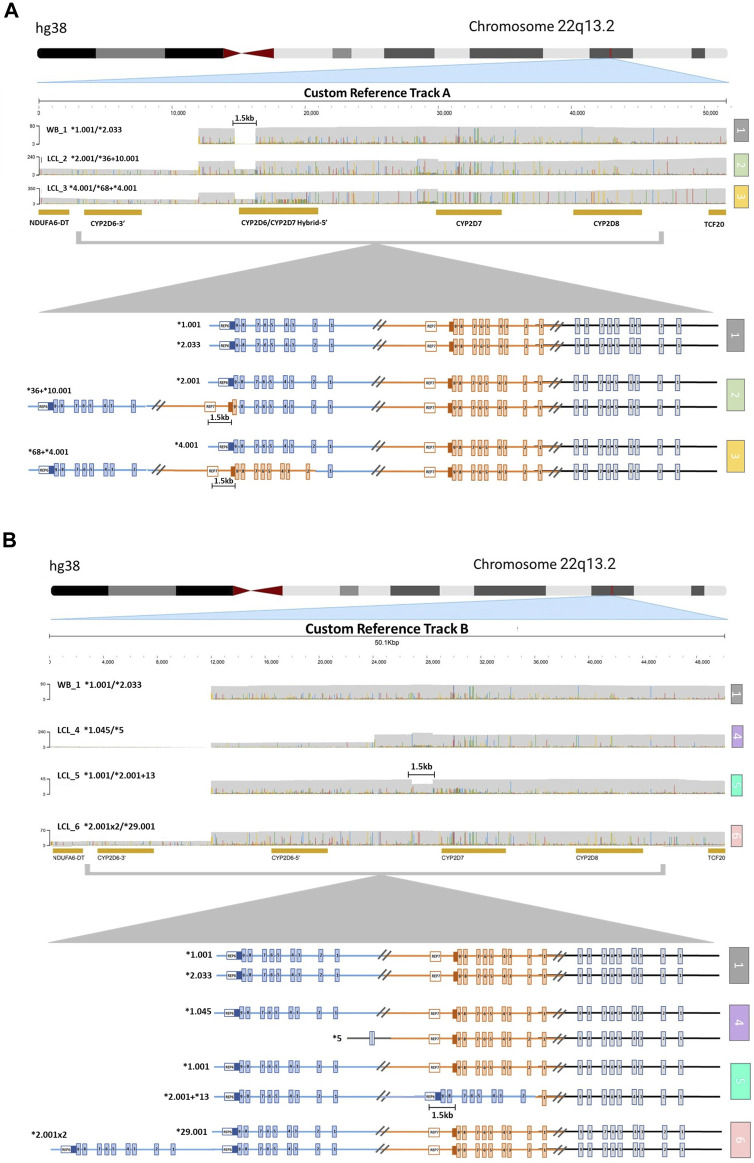
Visualization of *CYP2D6-2D7-2D8* Structural Variation. **(A)** Alignment to *CYP2D6/CYP2D7* hybrid duplication custom reference track **(A)** (1) Sample with no structural variation (SV) within the loci. The 1.5 kb long gap indicates the location of a *CYP2D7*-like spacer element that is only present in *CYP2D7-*derived downstream regions. (2) Sample with one allele (top) with no SV and one allele (bottom) containing a CYP2D6*36 in tandem with a CYP2D6*10. (3) Sample with one allele (top) with no SV and one allele (bottom) with a CYP2D6*68 hybrid in tandem with a CYP2D6*4. **(B)** Alignment to *CYP2D6* gene duplication custom reference track **(B)** (1) Sample with no SV. (4) Sample with one allele (top) with no SV and one allele (bottom) with the CYP2D6*5 gene deletion. (5) Sample with one allele (top) with no SV and one allele (bottom) with a CYP2D6*13 hybrid gene (exons two to nine and the downstream regions are derived from *CYP2D7*) in tandem with a CYP2D6*2. The 1.5 kb long gap indicates the location of a *CYP2D7*-like spacer element that is only present in *CYP2D7-*derived downstream regions. (6) Sample with one allele (top) with no SV and one allele (bottom) with a gene duplication (sample has two copies of CYP2D6*2).

The alignment of samples with SV/CNV to the custom reference tracks was done based on the nature of the 5′ region of *CYP2D6* (Supplementary Figure S1). Samples LCL_2 and LCL_3, which have SV/CNV that include a *CYP2D6-2D7* hybrid gene copy with *CYP2D7*-like 5′ region and spacer element (*36 and *68), were aligned to Custom Reference Track A (Figure 2A). Samples LCL_four to six, with SV/CNV that includes either a full *CYP2D6* gene duplication (*2x2), deletion (*5), or *CYP2D7-D6* hybrid gene copy (*2+*13) with a *CYP2D6* like 5′ region and no spacer element were aligned to Custom Reference Track B (Figure 2B).

Aligning to these custom reference sequences enabled the visualization of reads that ranged from ∼25 kb for samples with large *CYP2D6* deletions (Figure 2B, sample 4), up to ∼52 kb for samples with duplications or hybrid gene copies (Figure 2A, samples 2 and 3; Figure 2B, samples 5 and 6). Annotation of the custom reference tracks allowed for direct haplotype analysis of each allele containing an SV/CNV (Supplementary Table S3). We observed 100% concordance in diplotype calls between both technical replicates of LCL_6 when analyzed using the parameters listed above.

### 3.3 Characterization of *CYP2D7* and *CYP2D8* haplotypes

The full length reads covering the *CYP2D6-2D7-2D8* loci produced phased haplotypes not only for *CYP2D6,* but also for the highly polymorphic *CYP2D7* and *CYP2D8* pseudogenes. While *CYP2D6* is the only gene encoding a functional protein, it is important to understand genetic variation in *CYP2D7* and *CYP2D8* to fully characterize broader patterns of SV/CNV, as well as to interpret unusual genotypes or no calls, that can be caused by interfering variation in these pseudogenes (Gaedigk et al., 2015; Riffel et al., 2015).

Our analysis identified 17 *CYP2D7* and 18 *CYP2D8* unique haplotypes within our study data set. These haplotypes were comprised of 257 SNPs, 71 of which were in exons. Full-length sequencing of the loci also allowed us to determine full phased haplotype structure of the entire region (Supplementary Table S3) for all our samples, without employing a statistical inference model, independent of loci size, the nature of SV/CNV present, and/or sample DNA source.

## 4 Discussion

The genetic analysis of the clinically relevant *CYP2D6* gene is often complex and has presented substantial challenges to the testing community (Gaedigk, 2013; Hicks et al., 2014; Gaedigk et al., 2015; Riffel et al., 2015; Nofziger and Paulmichl, 2018; Nofziger et al., 2020). This has resulted in the potential of incorrect phenotype assignment, particularly in samples with less common haplotypes, SV/CNV, or from populations which have not been well characterized (Wang et al., 2022). Therefore, our goal was to develop an improved methodology to overcome these challenges through an approach that combines accurate SNP level genotyping with copy number analysis in one single assay utilizing LRS.

One potential benefit of LRS compared to traditional NGS is that less read depth has been shown to be required for SV/CNV characterization. As discussed by Stancu et al., mapping and phasing of structural variants was accurately done with only 11–16x depth in LRS WGS, compared to 35–40x depth often required for short read NGS (Chaisson et al., 2015; Shi et al., 2016; Cretu Stancu et al., 2017; Mantere et al., 2019; Leung et al., 2022; Zhou et al., 2022). As our enrichment and LRS analysis showed >35X coverage of the entire *CYP2D6-D7-D8* loci in all samples, we were able to determine both phased SNP level diplotypes and assign accurate *CYP2D6* copy number state.

By enriching the entire loci and any SV/CNV within it, we accurately genotyped all samples tested using one single methodology that generated libraries ready for sequencing in less than 1 day. Alternative approaches utilize either computational CNV assignment or can take multiple days to perform (e.g., short read NGS, WES, Sanger, microarray). Other approaches require multiple assays to determine both SV/CNV state (e.g., TaqMan Copy Number Assays) and SNP level genotypes (e.g., TaqMan SNP genotyping) and may not be able to determine which allele is the duplicated or hybrid allele. Another phenomenon that can impact accurate copy number detection is the presence of SNPs within a copy number assay probe or primer binding site that interfere with assay performance and generate false-positive calls for copy number loss (Turner et al., 2021). Our results were 100% concordant with existing *CYP2D6* genotype data and our analysis method provided further resolution of the specific suballeles present and resolved ambiguous phasing, which can impact correct phenotype assignment (Yang et al., 2017; Nofziger and Paulmichl, 2018; Erdmann et al., 2023)^,^ showing improvement over the results generated by the other methodologies (Tables 1, 2).

The impact of the high sequence similarity between *CYP2D6* and *CYP2D7* represents one of the main challenges for short read NGS sequencing in particular, as it relates to copy number analysis. It is well established that gene duplications, high sequence homology between genes, or the presence of pseudogenes substantially influences genotyping accuracy and sequence alignment. Short read studies using paired-end *CYP2D6* reads, have shown the underlying challenges with misaligning reads from *CYP2D6* to *CYP2D7* or *CYP2D8* (Twist et al., 2016; Yang et al., 2017). Longer read lengths can resolve misalignment in some cases, but the repetitive or highly similar regions like those located in the *CYP2D* loci still pose challenges. Sequencing of the entire loci using LRS can further reduce the misalignments and will allow for development of more accurate reference sequences, removing off target *CYP2D7* and *CYP2D8* misalignment in the highly similar regions. This can be of use for more accurate short read alignment and targeted assay development. This also removes any ambiguity of the location of structural events, and the need for computational assessment of copy number in the event of gene multiplications.

Further evaluation of *CYP2D7* and *CYP2D8* sequence data revealed additional findings. Of the exonic SNPs found in the *CYP2D7* and *CYP2D8* haplotypes, 59 had variant nucleotides corresponding to the reference nucleotide of *CYP2D6*, which may contribute to read misalignment(s) in short read NGS or other traditional genotyping approaches (Supplementary Table S3) (Pratt et al., 2016). Additionally, of the 59 SNPs found, ten have also been annotated as SNPs in *CYP2D6*. For example, rs61736524 (G>A) in exon 4 of *CYP2D8* matches rs748851484 (G>A) in exon 4 of *CYP2D6*. These SNPs may lead to false positive results with traditional SNP genotyping approaches such as TaqMan™ as seen with CYP2D6*15 and *35, where a SNP in *CYP2D7* matches the corresponding *CYP2D6* nucleotide, enabling primer binding and amplification from both genes and incorrect genotyping results (Riffel et al., 2015). This has previously only been reported in *CYP2D7* (Riffel et al., 2015; Scantamburlo et al., 2017), but not described in *CYP2D8,* though as shown by Gaedigk et al. for the *CYP2D6* *17 defining SNP rs28371706, both *CYP2D7* and *CYP2D8* share significant sequence similarity with *CYP2D6* in the SNP flanking region and the potential for off target genotyping (Gaedigk et al., 2015).

Taken together, these findings highlight the advantages of our enrichment approach compared to methodologies, which require multiple assays or approaches to fully characterize samples with *CYP2D6* SV/CNV (Huddleston et al., 2017; Yang et al., 2017; Nofziger and Paulmichl, 2018; Stephens et al., 2018; Bewicke-Copley et al., 2019; Mai et al., 2019; Saravanakumar et al., 2019; Amarasinghe et al., 2020; Gilpatrick et al., 2020; Erdmann et al., 2023).

## 5 Limitations and future work

Current *CYP2D6* annotation programs and star allele callers such as Aldy (Numanagić et al., 2018), StellarPGx (Twesigomwe et al., 2021), Cyrius (Chen et al., 2021), and Stargazer (Lee et al., 2019) are not optimized for handling long continuous reads for samples containing complex structural variation, and some, such as Stargazer rely existing allele databases which can lead to improper genotype assignment in populations that have not been well characterized (Twesigomwe et al., 2021; Wang et al., 2022).

Long read sequencing is facilitating improved *CYP2D6* genotyping, however, as shown by Mai et al. and others (Shi et al., 2016; Yang et al., 2017; Bewicke-Copley et al., 2019; Mai et al., 2019; Mantere et al., 2019; Erdmann et al., 2023), LRS is still hindered by aligning to current standard human reference genomes, such as GRCh37/38, which is based on samples from individuals of European ancestry and are often derived from short read sequence data (WES/WGS). This has led to misalignment, particularly in underrepresented populations and in highly similar or complex regions, like *CYP2D6* (Mai et al., 2019; Wang et al., 2022). While this study only includes a limited number of samples, we still found substantial sequence variation, which highlights the need to analyze additional samples from diverse populations to understand more fully and comprehensively the polymorphic nature of this complex gene locus and validate the clinical utility of the approach. To fully utilize the potential of LRS for *CYP2D6* genotyping, novel software programs will need to be developed that are able to use data aligned to references that include expanded annotation of SV/CNV for accurate variant calling in the SV containing regions.

## 6 Conclusion

Long read WGS can address some of the current limitations with *CYP2D6* genotyping, however highly complex regions still represent challenges for genome alignment, and clinical testing often requires a cost and time effective, and therefore targeted approach (Scantamburlo et al., 2017). The lower initial investment cost in nanopore sequencing (Cretu Stancu et al., 2017; Mantere et al., 2019; Leung et al., 2022; Zhou et al., 2022), and the ability to perform this assay in a clinically relevant turnaround time make this an attractive target for clinical use; however, advancements in sequencing quality and analysis software are still needed prior to clinical implementation. Here we provide proof-of-concept that our single-reaction, CRISPR-Cas9 based, PCR-free enrichment approach may overcome many of the limitations of current methods such as short read NGS, SNP-based genotyping, by directly capturing both SNP level variation and complex SV/CNV in a single assay, which can be performed using multiple clinically relevant sample types such as blood and saliva. In addition, as our approach captures the entire region in continuous long reads, data generated can be used to develop more accurate reference sequences and has the potential to improve alignment and more accurate genotype and phenotype assignment (Bu et al., 2020; Malekshoar et al., 2023).

## Data Availability

The datasets presented in this study can be found in online repositories. The names of the repository/repositories and accession number(s) can be found below: https://www.pharmvar.org/haplotype/126, https://www.pharmvar.org/haplotype/840, https://www.pharmvar.org/haplotype/1748, https://www.pharmvar.org/haplotype/129, https://www.pharmvar.org/haplotype/235, https://www.pharmvar.org/haplotype/179, https://www.pharmvar.org/haplotype/132, https://www.pharmvar.org/haplotype/222, https://www.pharmvar.org/haplotype/2303, https://www.pharmvar.org/haplotype/2304, https://www.pharmvar.org/haplotype/2305, https://a.storyblok.com/f/70677/x/4ba997d9db/cyp2d6_structural-variation_v2-6.pdf
