# Supplementary material for: Characterization of complex structural variation in the CYP2D6-CYP2D7-CYP2D8 gene loci using single-molecule long-read sequencing
Source: Front Pharmacol. 2023 Jun 22;14:1195778. doi: 10.3389/fphar.2023.1195778 (PMC10324673; doi:10.3389/fphar.2023.1195778)
Supplement: Supplementary file 2 [file Table1.DOCX]

Supplementary Material

**Characterization of Complex Structural Variation in the**

**CYP2D6-CYP2D7-CYP2D8 Gene Loci Using Single-molecule**

**Long-read Sequencing**

**Amy J Turner^1^, Ashley D Derezinski^1^, Andrea Gaedigk^2^, Mark E Berres^3^, David Gregornik^4^, Keith Brown^5^, Ulrich Broeckel^1^* and Gunter Scharer^1^***

^1^RPRD Diagnostics LLC, Milwaukee, WI, USA

^2^Children's Mercy Research Institute, Kanas City, MO, USA

^3^University of Wisconsin Madison Biotechnology Center, Madison, WI, USA

^4^Children's Minnesota, Minneapolis, MN, USA

^5^Jumpcode Genomics, San Diego, CA, USA

*** Correspondence:**Ulrich Broeckel and Gunter Scharer
UB: ubroeckel@rprdx.com. GS: gscharer@rprdx.com

# Supplementary Figures and Tables

**Table S1. crRNA Sequences.**

| **crRNA ID** | **Sequence** | **Genomic Location** | **Strand** |
| --- | --- | --- | --- |
| crRNA_1 | UGUCAAGAAUUAGUGGUGGU | Chr22: 42,123,054 | Positive |
| crRNA_2 | AAGGUGGUGGACACUCGUGA | Chr22: 42,161,339 | Negative |

Sequences listed 5’ to 3’.

**Table S2. Amplicon Primers Sequences**

| **Primer ID** | **Location** | **crRNA** |
| --- | --- | --- |
| Forward: AGACAGTAGTCCCAGTTGAGTC | Chr22: 42121845-42126954 | crRNA_1 |
| Reverse: ACCTGTCATCGGTGCTGAAG | Chr22: 42121845-42126954 | crRNA_1 |
| Forward: CCACGGGGAGTGGAAGGTA | Chr22:42155397-42162425 | crRNA_2 |
| Reverse: GAGGAGAGAAGCCCCTCCAG | Chr22:42155397-42162425 | crRNA_2 |

**Table S3. Summary of *CYP2D6, CYP2D7* and *CYP2D8* haplotypes.** See excel file.

##
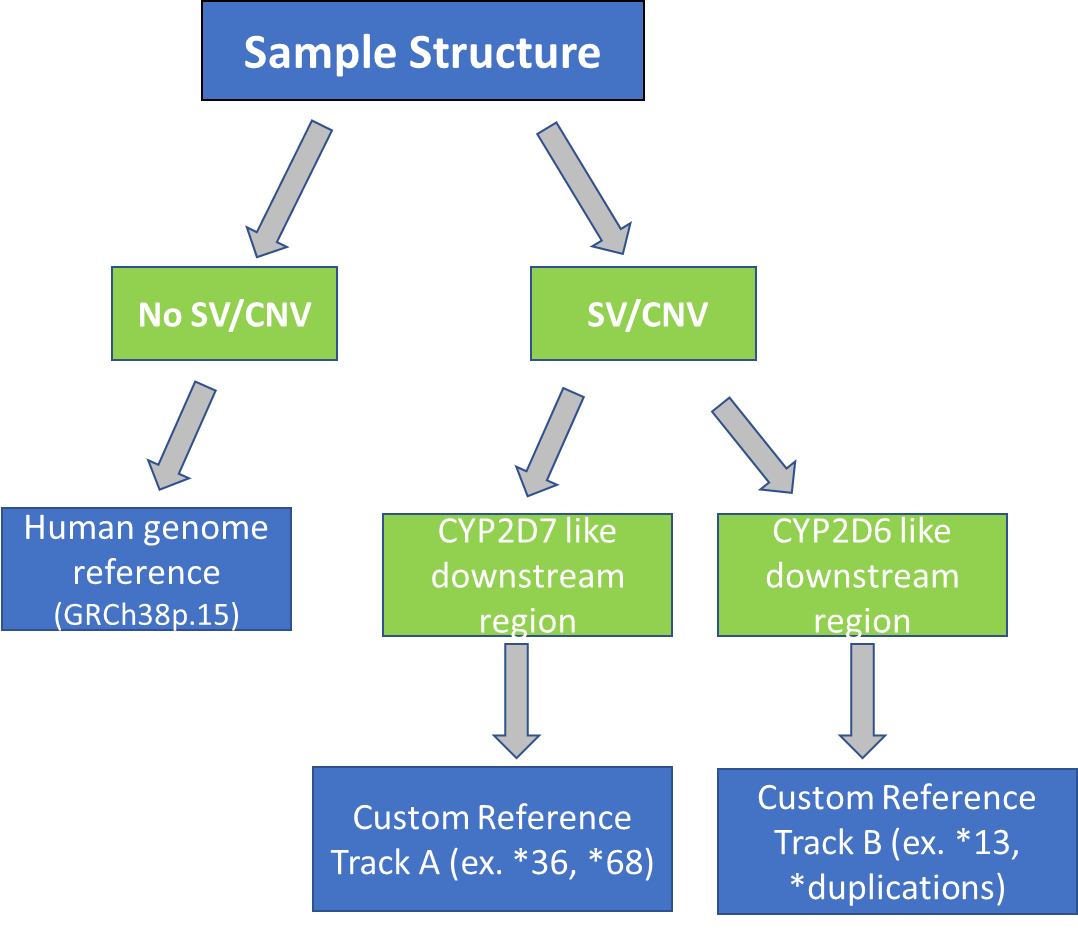
Supplementary Figures

**Supplemental Figure S1.** Reference selection workflow for samples with and without an SV/CNV. Samples with SV/CNV were aligned to each custom refence sequence based on the nature of the 5’ region of *CYP2D6* and the presence or absence of a *CYP2D7* like 1.56kb spacer element.

**
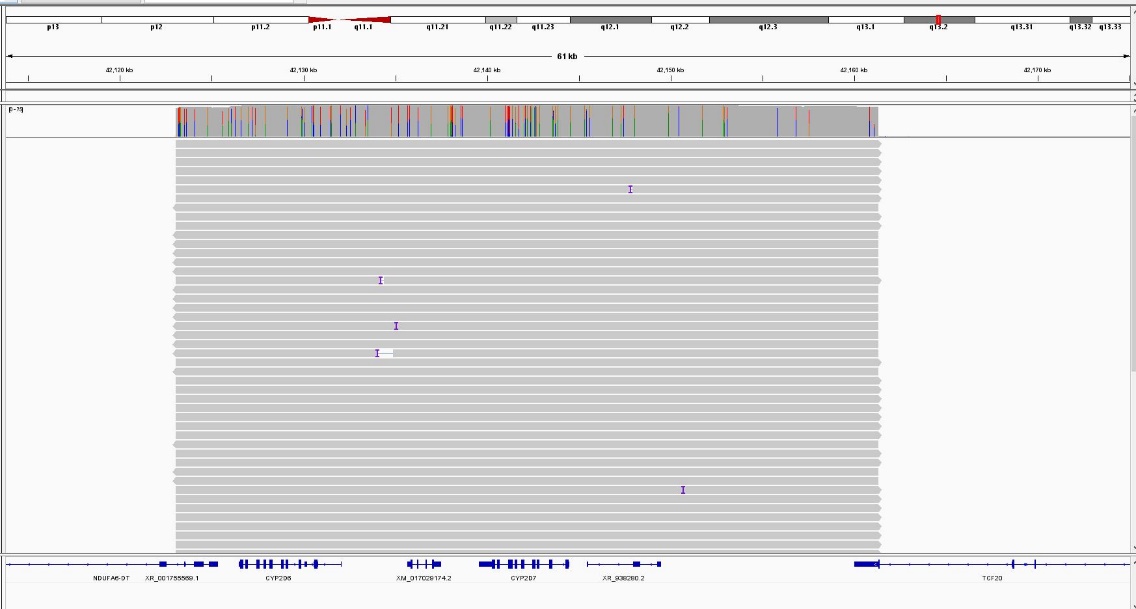
**

**Supplemental Figure S2.** Representative IGV alignment to hg38 of continuous 38kb reads obtained from a sample containing no structural variation and a read depth of >300x.
